# Evaluation of nutritional, anthropometric, and psychological outcomes in different metabolic and bariatric surgery techniques: a follow up study

**DOI:** 10.1186/s12893-025-02773-4

**Published:** 2025-01-25

**Authors:** Elham Hojaji, Zahra Veysi, Shadi Naderyan Fe’li, Neda Shalbaf, Moslem Arian, Cain C. T. Clark, Ahmad Reza Dorosty Motlagh

**Affiliations:** 1 Department of Community Nutrition, School of Nutritional Sciences and Dietetics, Tehran University of Medical Sciences, Tehran, Iran; 2 Department of Community Nutrition, Faculty of Nutrition Sciences and Food Technology, Shahid Beheshti University of Medical Sciences, Tehran, Iran; 3 Department of Epidemiology and Biostatistics, School of Public Health, Tehran University of Medical Sciences, Tehran, Iran; 4 Endocrine Research Center, Institute of Endocrinology and metabolism, department of internal medicine, school of medicine, Firoozgar Hospital, Iran University of medical sciences, Tehran, Iran; 5 Department of Counseling, University of Social Welfare and Rehabilitation Sciences, Tehran, Iran; 6 Center for Intelligent Healthcare, Coventry University, Coventry, CV1 5FB UK

**Keywords:** Obesity, Metabolic and bariatric surgery, Psychological problems, Dietary intake

## Abstract

**Background:**

Metabolic and bariatric surgery (MBS) is a suitable solution for the treatment of morbid obesity. Investigating an MBS method that has the best outcomes has always been the main concern of physicians. The current study aimed to compare nutritional, anthropometric, and psychological complications of individuals undergoing various MBS Techniques.

**Methods:**

A total of 96 subjects, who had been already referred to the obesity clinic of Firoozgar Hospital, Tehran, Iran, for MBS, were selected for the study and were followed for one year after MBS. The dietary intakes of the participants were assessed using a Food Frequency Questionnaire (FFQ) on a daily, weekly, monthly, or yearly basis. The mental health of participants was done via the Symptom Checklist 90 Revised. Furthermore, the In-Body 720 bioelectrical impedance device was used to obtain the body composition of the participants at the beginning and the end of the study.

**Results:**

The mean age of the participants was 39.5 *±* 9.5 years. All anthropometric indices including weight, Body Mass Index (BMI), protein content, fat mass, and fat mass percentage decreased after Roux-en-Y gastric bypass (RYGB), one-anastomosis gastric bypass (OAGB), and sleeve gastrectomy (SG) surgeries (*P* < 0.05). After adjustments for confounders, no significant difference was observed in the magnitude of the changes in body composition between the three Techniques (*p* > 0.05). Energy and carbohydrate intake significantly decreased after surgeries in all three groups (*p* < 0.05), but comparing the methods no significant difference was revealed (*p* > 0.05). Furthermore, the results indicated that there was a significant relationship between the mental health of patients before and after surgeries (*p* < 0.05).

**Conclusion:**

Overall, all three methods of surgery contributed to the improvement of the nutritional, anthropometric, and psychological complications. Further complementary studies are needed to determine the least complicated MBS method.

## Introduction

Obesity, a major health concern linked to non-communicable diseases, is characterized by excessive body fat and diagnosed using BMI (≥ 30 kg/m² for obesity, ≥ 40 kg/m² for morbid obesity). Its global prevalence is rising, with an estimated 38% of adults affected by 2030. In Iran, about 22.7% of adults are currently obese. Obesity, as a risk factor for many non-communicable diseases, represents one of the most important problems in the healthcare system [1].

Due to numerous obesity-related complications including hypertension, cardiovascular disease, and diabetes, management of obesity as a chronic condition is crucial [2]. Many therapeutic approaches have been investigated for the management of morbid obesity and its associated complications. Lifestyle modifications, calorie restriction, anti-obesity dietary drugs, and MBS are among the proposed strategies for weight management in obese subjects [3]. In many cases the lifestyle modifications are not appropriate, the MBS is recommended. MBS can effectively reduce weight and improve quality of life, resulting in sustained total body weight loss over many years for most patients [4, 5]. It is considered an MBS option for patients with BMI 35–40 kg/m2, or BMI 30–34.9 kg/m2 with obesity-related comorbidities [6]. 

The eating habits of patients undergoing MBS are one of the main factors that have been studied. The consumption of high-calorie and high-carbohydrate diets is reduced in such patients due to the new anatomical conditions of the gastrointestinal tract. The absorption of several minerals and vitamins might be disrupted. Although there has been evidence of increasing food intake and gastrointestinal system adaptation over time, malabsorption of nutrients may continue as a result of the structural changes [7–9]. Furthermore, a growing body of evidence has shown improvements in mental and psychological health after weight loss as a result of the surgery [10]. However, MBS could negatively affect depression and satisfaction of patients, depending on the amount of weight loss [11]. In addition, previous studies and observations have shown different changes in body weight and body composition based on the type of MBS, which some of them might be more successful in achieving their goal [12–14].

As mental and nutritional health status, alongside optimal body composition maintenance are considered important factors of a modified lifestyle, evaluation of such factors is necessary in these patients. Thus, this follow-up study was conducted to evaluate and compare the anthropometric, nutritional, and mental health status of subjects undergoing three surgical methods of MBS.

## Materials and methods

After obtaining ethics approval (IR.IUMS.REC 1396.31076), subjects were selected from patients of both sexes aged 18 to 65 who were referred to the obesity clinic and provided informed consent. Participants were categorized as either having grade III obesity (Body Mass Index [BMI] ≥ 40 kg/m²) or grade II obesity (BMI = 35–40 kg/m²) with accompanying conditions such as diabetes, hypertension, or other obesity-related diseases, qualifying them for MBS. The nutritional and mental health status of participants was assessed prior to surgery.

To calculate the sample size, the study by Torsten Olbers et al. [15] was reviewed. The minimum sample size was estimated to be 37 participants per group, considering a 95% confidence level and 90% power, based on the mean score for Total body fat (kg) in the mentioned study. For this purpose, the following command was entered in Stata software, and with the assumptions listed below, the sample size was estimated to be 37 participants for each group. We then increased the sample size to 40 participants for each of the surgery groups (SG, RYGB, and OAGB).

One-year post-MBS, 24 subjects were excluded from the study due to non-cooperation (Some patients were unable to participate in the study due to a lack of willingness to complete it, dissatisfaction with their surgical outcomes, lack of time to respond, or long distances from the study site), and the remaining patients underwent a follow-up assessment of their nutritional and mental health status. Individuals on psychiatric or antidepressant medications or those with cognitive impairments were excluded from the study.

Dietary intake was evaluated using a semi-quantitative food frequency questionnaire (FFQ) consisting of 148 common food items with standardized serving sizes. Participants reported the frequency of consumption for each item on a daily, weekly, monthly, or annual basis. The validity and reliability of the FFQ had been previously established [16]. Each food item was converted to grams based on home food guidance, and dietary intake analysis and nutrient calculation were conducted using software developed by the Endocrine Research Institute of Shahid Beheshti University of Medical Sciences [17].

The assessment of mental disorders utilized the SCL-90-R, a clinical symptoms questionnaire comprising 90 items rated on a 5-point Likert scale ranging from zero (not at all) to four (extremely). This scale encompasses nine dimensions, including somatization (12 items), obsessive-compulsive disorder (10 items), interpersonal sensitivity (9 items), depression (13 items), anxiety (10 items), hostility (6 items), phobia (7 items), paranoid ideation (6 items), psychosis (10 items), and seven additional items concerning sleep disorders and sexual desire. Mean values exceeding 1, 2, and 3 indicate psychiatric disorder, severe depression, and psychosis, respectively [18].

Also, physical activity was evaluated using the International Physical Activity Questionnaire – short form (IPAQ) [19]. Participants reported the duration and frequency of activities such as walking, moderate exercise, and vigorous sports over the week preceding the assessment. Physical activity levels were calculated in metabolic equivalent minutes per week, categorizing participants into sedentary, moderate, and active groups based on values of less than 600, 600–3000, and greater than 3000 MET/min/week, respectively.

Body composition was assessed using the In-Body 720 bioelectrical impedance device. Measurements included fat mass, total body fat percentage, visceral fat, muscle mass, and body protein content, evaluated at both the beginning and the end of the study. Patients self-reported their height and weight through a questionnaire, and BMI was calculated by dividing weight (kg) by height squared (m²).

To calculate the **Total Weight Loss Percentage (TWL%)** after bariatric surgery, we used the following formula:

$$\:TWL\%=\left(\frac{Initial\:Weight-Current\:Weight}{Initial\:Weight}\right)\times\:100$$

Statistical analyses were conducted using SPSS software (version 26). Descriptive statistics were computed with independent samples t-tests for age as a continuous variable, and chi-square tests for categorical and binary variables across the three Metabolic and Bariatric Surgery Techniques (SG, RYGB, and OAGB). Paired sample t-tests, analysis of covariance (ANCOVA), and McNemar tests were employed to evaluate primary and secondary outcomes, with *p*-values < 0.05 considered statistically significant. Furthermore, confounders including sex, age, family history of obesity, childhood obesity, marital status, smoking status, alcohol consumption, physical activity level, and education were adjusted using the ANCOVA test.

## Results

The mean average age of those 96 patients who enrolled in the study was 39.5 *±* 9.59 years. Thirty-four (35.4%), 37 (38.5%), and 25 (26%) subjects were respectively in the SG, OAGB, and bypass groups. The demographic characteristics of patients, TWL% are presented in Table 1.

**Table 1:** Baseline characteristics of participants, including total weight Loss% by type of metabolic and bariatric surgery

	SG(*n* = 34)	RYGB (*n* = 37)	OAGB (*n* = 25)	*p*-value*
**TWL%**	32.9 ± 8.1	36.7 ± 10.6	34.1 ± 8.2	0.22
**Age (years)**	40 ± 9.13	38.6 ± 10.2	38.2 ± 9.31	0.74
**Sex**				
Men	8 (23.5)	11 (29.7)	5 (20)	0.67
Women	26 (76.5)	26 (70.3)	20 (80)	
**Marital status**				
Married	21 (61.8)	27 (73)	17 (68)	0.60
Single	13 (38.2)	10 (27)	8 (32)	
**Education**				
< Diploma	7 (20.6)	9 (24.3)	3 (12)	0.48
≥Diploma	27 (79.4)	28 (75.7)	22 (88)	
**Smoking**				
Yes	11 (32.4)	14 (37.8)	11 (44)	0.66
No	23 (67.6)	23 (62.2)	14 (56)	
**Alcohol consumption**				
Yes	4 (11.8)	7 (18.9)	8 (32)	0.15
No	30 (88.2)	30 (81.1)	17 (68)	
**Physical activity level**				
Low	20 (58.9)	30 (81.1)	16 (64)	0.04
Medium	13 (38.2)	4 (10.8)	9 (36)	
High	1 (2.9)	3 (8.1)	0 (0)	
**Family history of obesity**				
Yes	29 (85.3)	33 (89.2)	17 (68)	0.08
No	5 (14.7)	4 (10.8)	8 (32)	
**Obesity in childhood**				
Yes	15 (44.1)	21 (56.8)	17 (68)	0.18
No	19 (55.9)	16 (43.2)	8 (32)	

Values are based on mean ± standard deviation or frequency (percentage). P -value less than 0.05 was considered significant. *One-way ANOVA and Chi-square test for quantitative and qualitative variables respectively. SG: Sleeve Gastrectomy. Roux-en-Y Gastric Bypass(RYGB). One-Anastomosis Gastric Bypass(OAGB)

The comparison of the mean and standard deviation of weight and body composition of patients is indicated in Table 2. The results show that except muscle mass for the RYGB and OAGB subjects, all other body composition indices were significantly reduced after MBS in all three methods (*p* < 0.05). After adjustments for various confounding variables, the RYGB surgery had a greater mean reduction in the BMI, body weight, fat mass, fat percentage, and body protein compared to the other two methods, but the results were non-significant (*p* > 0.05). Furthermore, subjects in the OAGB category had improved muscle mass (4.86 ± 7.81 kg) compared to the RYGB and SG surgery in a non-significant fashion (*p* = 0.56).

**Table 2:** Comparison of weight and body composition of patients

	SG(*n* = 34)	RYGB (*n* = 37)	OAGB (*n* = 25)	*P*-value^1^
**Body weight (kg)**	-40.5 ± 2.66	-49.3 ± 2.57	-44.0 ± 3.14	0.07
Before	117 ± 19.2	137 ± 25.3	124 ± 20.9	
After	78.7 ± 16.7	85.2 ± 15.6	81.2 ± 11.6	
*p*-value^2^	< 0.001	< 0.001	< 0.001	
**BMI (kg/m**^2^)	-18.2 ± 1.86	-19.6 ± 1.76	-17.6 ± 2.16	0.75
Before	42.9 ± 3.49	49.2 ± 7.84	46.3 ± 6.77	
After	28.7 ± 4.88	30.1 ± 5.26	29.8 ± 4.18	
*p*-value^2^	< 0.001	< 0.001	< 0.001	
**Body fat mass (kg)**	-31.0 ± 2.16	-37.4 ± 2.08	-32.5 ± 2.55	0.10
Before	56.3 ± 9.29	67.2 ± 13.4	60.6 ± 14.1	
After	26.4 ± 10.6	28.1 ± 12.0	29.0 ± 8.82	
*p*-value^2^	< 0.001	< 0.001	< 0.001	
**Fat mass percentage**	-16.1 ± 1.43	-17.3 ± 1.38	-16.2 ± 1.69	0.83
Before	49.1 ± 3.95	50.1 ± 4.28	50.8 ± 7.95	
After	33.6 ± 9.19	31.9 ± 10.1	35.1 ± 8.99	
*p*-value^2^	< 0.001	< 0.001	< 0.001	
**Muscle mass (kg)**	-6.03 ± 6.63	0.61 ± 6.39	4.86 ± 7.81	0.56
Before	39.2 ± 8.10	38.3 ± 10.2	33.6 ± 8.25	
After	28.1 ± 7.41	37.7 ± 40.8	38.5 ± 49.8	
*p*-value^2^	< 0.001	0.94	0.63	
**Body protein (kg)**	-1.62 ± 0.33	-2.14 ± 0.32	-1.79 ± 0.39	0.54
Before	11.5 ± 2.70	13.3 ± 3.38	11.8 ± 2.75	
After	10.2 ± 2.65	10.9 ± 2.21	10.1 ± 1.95	
*p*-value^2^	< 0.001	< 0.001	< 0.001	
**Waist-to-hip ratio**	-0.12 ± 0.02	-0.12 ± 0.02	-0.13 ± 0.02	0.95
Before	1.02 ± 0.03	1.02 ± 0.06	1.03 ± 0.13	
After	0.90 ± 0.06	0.89 ± 0.07	0.91 ± 0.06	
*p*-value^2^	< 0.001	< 0.001	< 0.001	

Values are based on mean ± standard deviation. P -value less than 0.05 was considered significant. ^1^*p*-values Obtained from ANCOVA. ^2^*p*-values obtained from paired sample t-test. SG: Sleeve Gastrectomy. Roux-en-Y Gastric Bypass(RYGB). One-Anastomosis Gastric Bypass(OAGB)

The comparison of the mean and standard deviation of dietary macronutrient intake in patients is indicated in Table 3. Energy and carbohydrate, mono-unsaturated fatty acids (MUFA), Phosphorus intake significantly decreased after surgeries in all three groups. Protein, total fat, saturated fatty acids (SFA), polyunsaturated fatty acids (PUFA), and cholesterol intake decreased significantly in the RYGB group. In addition, cholesterol intake were significantly reduced after SG bypass surgeries (*p* < 0.05). Micronutrients such as vitamin C, D,K, riboflavin, and cobalamin were significantly reduced only after RYGB surgery. Also, the mean intake of vitamin E, thiamine, niacin, folate, magnesium, copper, and selenium before and after the RYGB, and OAGB surgeries were significantly different. The mean intake of fiber, pyridoxine, zinc, and manganese was reduced significantly only after the OAGB surgery. After controlling for potential confounders, there is no significant difference in dietary intake between the study groups. After one year of MBS, the macronutrient intake in the SG group decreased more compared to the other two groups, non-significantly (*P* > 0.05). Calcium, manganese, and in the SG group had a larger decrease compared to the other groups, while phosphorus, zinc, copper, and total fiber in the RYGB group had a greater decrease compared to the other groups. In addition, total energy, iron, magnesium, vitamin D, vitamin E, vitamin K, and group B vitamins in the OAGB group had the largest decrease compared to the other groups. However, these results were not statistically significant (*P* > 0. 05). Interestingly, the intake of the vitamin A, vitamin C, vitamin D, vitamin E, vitamin K, and B vitamins except folic acid and biotin in the SG surgery, had the most increasing trend (*P* > 0.05).

**Table 3:** The comparison of daily dietary macronutrient intake of patients

	SG(*n* = 34)	RYGB (*n* = 37)	OAGB (*n* = 25)	*P*-value^1^
**Energy (kcal)**	-752 ± 94.3	-718 ± 90.8	-782 ± 111	0.91
Before	2535 ± 574	2488 ± 399	2600 ± 424	
After	1739 ± 247	1796 ± 225	1841 ± 312	
*p*-value^2^	< 0.001	< 0.001	< 0.001	
**Protein (g)**	-50.7 ± 79.8	-13.5 ± 76.8	-37.2 ± 93.9	0.95
Before	197 ± 656	85.7 ± 17.7	82.8 ± 17.0	
After	130 ± 365	69.1 ± 10.2	72.9 ± 15.0	
*p*-value^2^	0.61	< 0.001	0.05	
**Carbohydrates (g)**	-95.3 ± 14.2	-84.9 ± 13.7	-93.9 ± 16.8	0.86
Before	333 ± 77.1	321 ± 67.2	339 ± 71.7	
After	225 ± 60.0	246 ± 38.0	248 ± 45.6	
*p*-value^2^	< 0.001	< 0.001	< 0.001	
**Total fat (g)**	-72.8 ± 30.6	-36.3 ± 29.5	-47.6 ± 36.1	0.69
Before	147 ± 279	101 ± 24.8	108 ± 28.1	
After	68.7 ± 47.2	65.5 ± 11.9	67.7 ± 16.1	
*p*-value^2^	0.12	< 0.001	< 0.001	
**Cholesterol (g)**	-82.2 ± 22.5	-73.4 ± 21.7	-30.6 ± 26.6	0.32
Before	265 ± 105	256 ± 105	240 ± 121	
After	180 ± 65.4	188 ± 37.5	205 ± 68.4	
*p*-value^2^	< 0.001	0.001	0.28	
**SFA (g)**	-42.4 ± 21.3	-13.2 ± 20.5	-15.0 ± 25.0	0.57
Before	66.1 ± 200	29.6 ± 10.3	30.3 ± 9.83	
After	19.2 ± 10.0	17.7 ± 3.34	19.8 ± 4.59	
*p*-value^2^	0.18	< 0.001	< 0.001	
**MUFA (g)**	-22.8 ± 6.27	-14.7 ± 6.04	-17.8 ± 7.39	0.66
Before	42.0 ± 54.9	34.5 ± 10.88	38.5 ± 12.4	
After	18.8 ± 6.76	19.5 ± 5.59	21.7 ± 7.19	
*p*-value^2^	0.02	< 0.001	0.001	
**PUFA (g)**	-89.1 ± 57.3	-11.8 ± 55.2	-25.1 ± 67.6	0.61
Before	113 ± 543	21.7 ± 8.09	23.9 ± 9.35	
After	11.7 ± 6.02	11.5 ± 4.21	12.9 ± 5.01	
*p*-value^2^	0.28	< 0.001	< 0.001	
**Total fiber (g)**	37.0 ± 115	-87.7 ± 111	-22.1 ± 136	0.75
Before	91.8 ± 288	163 ± 709	44.4 ± 16.2	
After	163 ± 747	35.4 ± 9.80	34.0 ± 6.34	
*p*-value^2^	0.61	0.28	0.01	
**Calcium (mg)**	-21.9 ± 99.3	-6.82 ± 95.7	-18.1 ± 117	0.99
Before	1254 ± 409	1194 ± 328	1255 ± 315	
After	1225 ± 473	1211 ± 393	1210 ± 357	
*p*-value^2^	0.80	0.85	0.50	
**Iron (mg)**	-9.88 ± 38.7	8.56 ± 37.3	-18.2 ± 45.6	0.90
Before	89.6 ± 314	34.2 ± 15.1	39.1 ± 14.1	
After	74.2 ± 184	39.7 ± 22.6	33.08 ± 18.2	
*p*-value^2^	0.81	0.22	0.21	
**Zinc (mg)**	5.32 ± 19.0	-44.4 ± 18.3	-3.10 ± 22.4	0.15
Before	11.9 ± 2.70	57.1 ± 160.6	12.3 ± 3.25	
After	19.5 ± 51.2	9.72 ± 1.94	10.4 ± 2.18	
*p*-value^2^	0.40	0.08	0.01	
**Phosphorus (mg)**	-214 ± 81.2	-304 ± 78.2	-187 ± 95.7	0.60
Before	1440 ± 404.	1502 ± 363	1537 ± 430	
After	1191 ± 309	1234 ± 150	1344 ± 272	
*p*-value^2^	0.01	< 0.001	0.04	
**Magnesium (mg)**	-32.9 ± 26.2	-55.6 ± 25.9	-81.3 ± 30.5	0.50
Before	396 ± 121	404 ± 98.6	448 ± 159	
After	357 ± 89.9	355 ± 59.2	366 ± 70.7	
*p*-value^2^	0.20	0.01	0.02	
**Manganese (mg)**	-92.5 ± 78.2	3.92 ± 75.3	-24.6 ± 92.1	0.68
Before	8.09 ± 3.42	8.11 ± 3.38	10.9 ± 4.92	
After	8.00 ± 2.34	8.06 ± 2.29	8.04 ± 2.31	
*p*-value^2^	0.42	0.85	0.01	
**Copper (mg)**	-0.10 ± 0.45	-1.31 ± 0.44	-0.43 ± 0.53	0.16
Before	1.63 ± 0.43	2.64 ± 3.82	1.65 ± 0.43	
After	1.50 ± 1.11	1.31 ± 0.24	1.32 ± 0.21	
*p*-value^2^	0.70	0.04	0.01	
**Selenium (mcg)**	-0.10 ± 0.45	-1.31 ± 0.44	-0.43 ± 0.53	0.16
Before	111 ± 44.2	111 ± 44.2	144 ± 65.7	
After	85.2 ± 23.3	85.2 ± 23.3	94.9 ± 27.7	
*p*-value^2^	0.30	< 0.001	< 0.001	
**Vitamin A (RAE)**	175 ± 72.9	26.1 ± 70.3	31.0 ± 85.9	0.29
Before	774 ± 520	645 ± 255	623 ± 216	
After	604 ± 193	615 ± 178	591 ± 166	
*p*-value^2^	0.72	0.18	0.41	
**Vitamin C (mg)**	30.1 ± 17.0	26.1 ± 16.4	15.3 ± 20.0	0.85
Before	133 ± 58.4	110 ± 45.2	105 ± 34.1	
After	159 ± 106	142 ± 60.8	118 ± 50.4	
*p*-value^2^	0.22	0.03	0.32	
**Vitamin D (mcg)**	109 ± 96.8	54.1 ± 93.2	-27.6 ± 114	0.67
Before	16.5 ± 84.6	1.82 ± 1.22	2.02 ± 1.41	
After	165 ± 944	1.33 ± 0.83	1.94 ± 1	
*p*-value	0.37	0.04	0.91	
**Vitamin E (mg)**	100 ± 83.1	42.5 ± 80.0	-28.2 ± 97.9	0.62
Before ^2^	13 ± 14.3	14.5 ± 5.1	15.8 ± 5.8	
After	149 ± 819.1	9.1 ± 3	9.7 ± 3.5	
*p*-value^2^	0.34	< 0.001	< 0.001	
**Vitamin K (mcg)**	129 ± 75.2	145 ± 72.5	-8.48 ± 88.6	0.38
Before	344 ± 244	325 ± 227	393 ± 210	
After	504 ± 364	459 ± 330	361 ± 274	
*p*-value^2^	0.06	0.04	0.66	
**Thiamin (mg)**	83.2 ± 66.4	37.3 ± 64.0	-18.3 ± 78.2	0.62
Before	2.22 ± 1.39	2.03 ± 0.58	2.23 ± 0.72	
After	114 ± 653	1.45 ± 0.28	1.51 ± 0.28	
*p*-value^2^	0.33	< 0.001	< 0.001	
**Riboflavin (mg)**	20.2 ± 19.9	10.7 ± 19.1	-6.01 ± 23.4	0.70
Before	6.91 ± 28.4	1.89 ± 0.47	2.00 ± 0.42	
After	94.7 ± 192	1.57 ± 0.41	1.81 ± 0.42	
*p*-value^2^	0.41	0.02	0.14	
**Niacin (mg)**	20.2 ± 19.9	10.7 ± 19.1	-6.01 ± 23.4	0.70
Before	23.9 ± 6.22	25.2 ± 5.18	25.4 ± 5.52	
After	98.7 ± 467	19.5 ± 3.84	19.3 ± 4.33	
*p*-value^2^	0.36	< 0.001	< 0.001	
**Pyridoxine (mg)**	2.86 ± 2.77	1.44 ± 2.67	-1.15 ± 3.26	0.65
Before	2.92 ± 6.03	1.77 ± 0.41	1.91 ± 0.33	
After	6.82 ± 26.2	1.72 ± 0.32	1.72 ± 0.3	
*p*-value^2^	0.41	0.33	0.02	
**Folic acid (mcg)**	-66.6 ± 32.5	-89.5 ± 31.3	-123 ± 38.2	0.55
Before	572 ± 155	583 ± 117	602 ± 140	
After	501 ± 218	490 ± 86.8	490 ± 82.8	
*p*-value^2^	0.11	< 0.001	0.002	
**Cobalamin (mcg)**	63.0 ± 33.3	18.8 ± 32.1	-12.0 ± 39.2	0.34
Before	3.83 ± 1.67	3.61 ± 1.52	3.22 ± 1.36	
After	79.5 ± 325	2.64 ± 1.02	3.21 ± 1.14	
*p*-value^2^	0.18	0.01	0.9	
**Biotin (mcg)**	-4.57 ± 3.24	-3.19 ± 3.12	-6.50 ± 3.82	0.81
Before	30 ± 23.8	27.1 ± 10.1	30.7 ± 15.2	
After	24.1 ± 7.45	24.3 ± 4.16	25.4 ± 5.67	
*p*-value^2^	0.17	0.15	0.1	

Values are based on mean ± standard deviation. P -value less than 0.05 was considered significant. ^1^*P*-values obtained from ANCOVA. ^2^*P*-values obtained from paired sample t-test. SG: Sleeve Gastrectomy. Roux-en-Y Gastric Bypass(RYGB). One-Anastomosis Gastric Bypass(OAGB)

In Table 4, the comparison of psychological disorders is presented. There was a significant difference in the mental health status of patients before and after surgeries.

**Table 4:** Comparison of psychological disorders before and after of three methods of metabolic and bariatric surgery

Type of surgery	Psychotic disorder before surgery		Psychotic disorder after surgery		*p*-value
	No	Yes	No	Yes	
SG	31 (36.5)	3 (27.3)	33 (37.5)	1 (12.5)	0.625
RYGB	32 (37.6)	5 (45.5)	31 (35.2)	6 (75.0)	1.00
OAGB	22 (25.9)	3 (27.3)	24 (27.3)	1 (12.5)	0.50
**Type of surgery**	**Paranoid ideation disorder before surgery**		**Paranoid ideation disorder after surgery**		
	**No**	**Yes**	**No**	**Yes**	
SG	24 (42.1)	10 (25.6)	24 (40.0)	10 (27.8)	1.00
RYGB	21 (36.8)	16 (41.0)	23 (38.3)	14 (38.9)	0.687
OAGB	12 (21.1)	13 (33.3)	13 (21.7)	12 (33.3)	1.00
**Type of surgery**	**Phobia before surgery**		**Phobia after surgery**		
	**No**	**Yes**	**No**	**Yes**	
SG	31 (37.8)	3 (21.4)	30 (34.5)	4 (44.4)	1.00
RYGB	32 (39.0)	5 (35.7)	36 (41.4)	1 (11.1)	0.125
OAGB	19 (23.2)	6 (42.9)	21 (24.1)	4 (44.4)	0.50
**Type of surgery**	**Hostility before surgery**		**Hostility after surgery**		
	**No**	**Yes**	**No**	**Yes**	
SG	20 (33.3)	14 (38.9)	26 (36.1)	8 (33.3)	0.180
RYGB	24 (40.0)	13 (36.1)	26 (36.1)	11 (45.8)	0.754
OAGB	16 (26.7)	9 (25.0)	20 (27.8)	5 (20.8)	0.125
**Type of surgery**	**Anxiety before surgery**		**Anxiety after surgery**		
	**No**	**Yes**	**No**	**Yes**	
SG	25 (37.3)	9 (31.0)	28 (37.3)	6 (28.6)	0.453
RYGB	24 (35.8)	13 (44.8)	27 (36.0)	10 (47.6)	0.375
OAGB	18 (26.9)	7 (24.1)	20 (26.7)	5 (23.8)	0.625
**Type of surgery**	**Depression before surgery**		**Depression after surgery**		
	**No**	**Yes**	**No**	**Yes**	
SG	23 (37.7)	11 (31.4)	29 (39.2)	5 (22.7)	0.146
RYGB	21 (34.4)	16 (45.7)	25 (33.8)	12 (54.5)	0.344
OAGB	17 (27.9)	8 (22.9)	20 (27.0)	5 (22.7)	0.250
**Type of surgery**	**Interpersonal sensitivity before surgery**		**Interpersonal sensitivity after surgery**		
	**No**	**Yes**	**No**	**Yes**	
SG	23 (37.7)	11 (31.4)	27 (36.0)	7 (33.3)	0.388
RYGB	23 (37.7)	14 (40.0)	30 (40.0)	7 (33.3)	0.039
OAGB	15 (24.6)	10 (28.6)	18 (24.0)	7 (33.3)	0.250
**Type of surgery**	**Obsessive compulsive before surgery**		**Obsessive compulsive after surgery**		
	**No**	**Yes**	**No**	**Yes**	
SG	19 (35.2)	15 (35.7)	26 (40.0)	8 (25.8)	0.039
RYGB	19 (35.2)	18 (42.9)	25 (38.5)	12 (38.7)	0.070
OAGB	16 (29.6)	9 (21.4)	14 (21.5)	11 (35.5)	0.687
**Type of surgery**	**Somatization before surgery**		**Somatization after surgery**		
	**No**	**Yes**	**No**	**Yes**	
SG	22 (38.6)	12 (30.8)	27 (40.9)	7 (23.3)	0.227
RYGB	21 (36.8)	16 (41.0)	24 (36.4)	13 (43.3)	0.453
OAGB	14 (24.6)	11 (28.2)	15 (22.7)	10 (33.3)	1.00

Values are based on frequency (percentage). *P*-values obtained from McNemar test for comparison of psychological disorders before and after surgery (frequencies were not reported). P -value less than 0.05 was considered significant. SG: Sleeve Gastrectomy. Roux-en-Y Gastric Bypass(RYGB). One-Anastomosis Gastric Bypass(OAGB)

Frequency of interpersonal sensitivity in RYGB group was significantly greater before surgery than after surgery (*p* = 0.039). Moreover, frequency of obsessive compulsive in the SG group was significantly greater before surgery than after surgery (*p* = 0.039).

Additionally, there were no significant differences in psychological disorders among the various MBS groups (*P* > 0.05).

## Discussion

In this follow-up study involving 96 patients who underwent bariatric surgery, we examined the differences among three surgical methods— RYGB, OAGB, and SG surgeries —regarding anthropometric changes, nutritional outcomes, and psychological status.

### Anthropometric changes

The study found that while the mean Total Weight Loss percentage (TWL%) was highest in the RYGB group, followed by OAGB, and lowest in SG, there was no statistically significant difference among the three groups (*p* > 0.05). Literature reveals mixed results: some studies suggest OAGB achieves higher Excess Body Weight Loss (%EBWL) compared to SG, especially in the first 1–2 years, while others report SG shows faster weight loss initially but OAGB provides better outcomes after five years [20, 21]. Overall, all three procedures lead to significant weight loss, with individual factors like initial BMI and comorbidities influencing outcomes [22].

Our findings show that except muscle mass for the RYGB and OAGB subjects, all other body composition indices were significantly reduced after all three methods (*p* < 0.05). After adjustments for various confounding variables, the RYGB surgery had a greater mean reduction in the BMI, body weight, fat mass, fat percentage, and body protein compared to the other two methods, but the results were non-significant (*p* > 0.05). Furthermore, subjects in the OAGB category had improved muscle mass compared to the RYGB and SG surgery in a non-significant fashion (*p* = 0.56).

The decreased degree of fat mass, body protein, and waist to hip ratio after the first year of the surgical procedure varies among bariatric surgery patients and is influenced by multiple factors, such as dietary protein intake, age, exercise, and mobility [23, 24].

Comparative studies suggest that while RYGB leads to greater overall weight loss compared to SG, it may compromise muscle mass retention. In contrast, SG tends to better preserve muscle mass. [25–27]. Additionally, another study demonstrated that in patients with a BMI greater than 50 kg/m², RYGB provided more effective weight reduction than SG [27].

### Nutritional outcomes

Our findings revealed no significant differences among the three surgical techniques in terms of the adjusted mean intake of energy, macronutrients, and micronutrients. This result aligns with prior studies suggesting comparable nutritional outcomes across different bariatric procedures.

Total energy and carbohydrate intake decreased significantly in all groups post-surgery. As shown in another study, in 66% of the patients tested, a significant reduction in carbohydrate intake occurred after surgery [28], which was concordant with the results of our study. However, in a meta-analysis study, the authors found no significant impact of MBS methods, including RYGB, and SG, on carbohydrate intake compared with baseline values [29].

Specific to SG, we noted a marked reduction in cholesterol and monounsaturated fatty acid (MUFA) intake, suggesting potential benefits in improving lipid profiles. In consistent a study showed that bariatric surgery, particularly SG, leads to significant changes in dietary consumption, including reductions in cholesterol and unsaturated fatty acid intake [30]. It could be suggested that the increased consumption of dairy products, fruits, and vegetables, along with a reduction in the intake of fats, oils, bread, and cereals, may be the reason for the decreased cholesterol intake in patients undergoing SG [31].

For RYGB, a significant decrease in protein, total fat, and polyunsaturated fatty acid (PUFA) intake was observed. In line with our findings, in one study there was a significantly lower level of total FA from the time of admission to 1 year after bariatric surgery [32].

Stefater et al. also highlighted that post-surgical patients often face challenges in meeting their nutritional needs, particularly concerning food intake. Their study indicated that inadequate dietary fat could lead to deficiencies in fat-soluble vitamins, which are crucial for overall health [33]. 

Conversely, some studies have reported that not all patients experience significant decreases in protein and fat intake post-surgery. For instance, Andreu et al. (2017) found that while some patients undergoing laparoscopic Roux-en-Y gastric bypass (LGBP) or sleeve gastrectomy (LSG) did experience reduced protein intake, others maintained adequate levels, suggesting variability in dietary adherence and individual responses to surgery [34]. To effectively manage protein and fat intake, tailored nutritional strategies are essential. This approach helps reduce the risk of deficiencies and improves patient outcomes, particularly in maintaining lean mass and overall health [35]. 

### Psychological changes

This study found no significant psychological changes across the three bariatric surgery groups based on the SCL-90-R questionnaire, except for a significant reduction in interpersonal sensitivity in the RYGB group (*p* = 0.039) and a decrease in obsessive-compulsive tendencies in both the RYGB (*p* = 0.070) and SG groups (*p* = 0.039). These psychological improvements can be attributed to multiple factors, including hormonal changes induced by surgery and improvements in body image and etc.

It can be said that bariatric surgery leads to reduced interpersonal sensitivity for several key reasons. For example, a systematic review by Herpertz and colleagues [36] found that mental health and psychosocial status, including social relations, improve for most people after bariatric surgery. This improvement in social relations can decrease interpersonal sensitivity as individuals become more confident and socially active [37]. Additionally, a longitudinal study by Demeireles and colleagues [38] showed significant improvements in body image and psychological well-being one year after surgery. This boost in body image can enhance self-esteem, leading to fewer feelings of social inadequacy and reduced sensitivity in interpersonal interactions. Similarly, Behrens and colleagues [39] noted that bariatric surgery triggers complex behavioral, physiological, and cognitive changes that improve both health and psychosocial outcomes. These changes, particularly the improvements in body image, are critical in reducing interpersonal sensitivity as they help individuals feel better about themselves and more secure in social settings. These psychosocial improvements are not solely due to weight loss but are significantly influenced by the enhanced body image and the resulting positive self-perception, contributing to decreased interpersonal sensitivity.

Additionally, Weight loss can reduce some obsessive behaviors related to eating and body image. As the study by Pyykkö [40] demonstrates, weight loss was associated with greater improvement in health-related quality of life, self-efficacy to exercise and controlling eating behaviors, self-esteem, and greater amelioration in food cravings. Furthermore, the surgery-induced hormonal shifts, particularly increases in glucagon-like peptide-1 (GLP-1), peptide YY (PYY), and reductions in ghrelin levels, have been shown to positively affect mood, reduce anxiety, and alleviate obsessive-compulsive symptoms [41–44]. Therefore, it can be said that overall, bariatric surgery leads to improvements in body image, self-perception, and relationships with others, resulting in reduced interpersonal sensitivity towards body and self-image. All these factors have complex interrelations and can be further explored for causal analysis. However, some research indicates no significant changes in interpersonal sensitivity post-surgery, suggesting additional interventions may be required [45]. Moreover, the psychological effects vary between surgical methods, with some procedures, like SG, showing limited impact on obsessive-compulsive tendencies [46, 47]. The reasons why these changes were more pronounced in certain groups need further investigation.

### Strengths and limitations

This study provides novel insights by evaluating the nutritional, anthropometric, and psychological impacts of three bariatric surgery techniques within an Iranian population. Data collection by qualified clinicians using validated tools ensures reliability. However, limitations include a reduced sample size, which may affect the precision of findings, and minor biases in dietary assessments due to recall bias and misclassification errors inherent in the use of the Food Frequency Questionnaire (FFQ).

Another limitation of the study was the lack of control over the use of medications for managing psychological disorders and dietary supplements by the patients. However, before surgery, patients were generally asked whether they had a history of using dietary supplements or were currently consuming them. Furthermore, this study investigated the trends in anthropometric, nutritional, and psychological changes up to one year after bariatric surgery. Consequently, future studies with a longer follow-up period are recommended.

## Conclusion

The findings of this study highlight both the benefits and challenges associated with different bariatric surgery techniques. While the surgeries lead to significant improvements in weight and certain psychological parameters, careful monitoring of nutritional intake is crucial to prevent deficiencies and ensure long-term health outcomes. Future studies with larger sample sizes and extended follow-ups are warranted to confirm and expand upon these results.

## Data Availability

The datasets generated and analyzed during the current study are available from the corresponding authors upon reasonable request.

## Abbreviations

BMI: Body mass index
RYGB: Roux-en-Y gastric bypass
OAGB: One-anastomosis gastric bypass
SG: Sleeve gastrectomy
LGBP: Laparoscopic roux-en-Y gastric bypass
LSG: Laparoscopic sleeve gastrectomy
MBS: Metabolic and bariatric surgery
FFQ: Food frequency questionnaire
IPAQ: International physical activity questionnaire
TWL: Total weight loss
EBWL: Excess body weight loss
GLP-1: Glucagon-like peptide-1
PYY: Peptide YY
PUFA: Polyunsaturated fatty acid
MUFA: Mono-unsaturated fatty acids
SFA: Saturated fatty acids

## References

[1] Azadnajafabad S, Mohammadi E, Aminorroaya A, Fattahi N, Rezaei S, Haghshenas R, et al. Non-communicable diseases’ risk factors in Iran; a review of the present status and action plans. Journal of Diabetes & Metabolic Disorders; 2021. 10.1007/s40200-020-00709-8 PMC7821170 33500879

[2] Valencia WM, Stoutenberg M, Florez H. Weight loss and physical activity for disease prevention in obese older adults: an important role for lifestyle management. Curr Diab Rep. 2014;14:1–10. 10.1007/s11892-014-0539-4 25183491

[3] Baker JS, Supriya R, Dutheil F, Gao Y. Obesity: treatments, conceptualizations, and future directions for a growing problem. Biology. 2022;11(2):160. 35205027 10.3390/biology11020160 PMC8869388

[4] Brunault P, Jacobi D, Léger J, Bourbao-Tournois C, Huten N, Camus V, et al. Observations regarding ‘quality of life’and ‘comfort with food’after bariatric surgery: comparison between laparoscopic adjustable gastric banding and sleeve gastrectomy. Obes Surg. 2011;21(8):1225. 21533881 10.1007/s11695-011-0411-4

[5] Aminian A, Wilson R, Al-Kurd A, Tu C, Milinovich A, Kroh M, et al. Association of bariatric surgery with Cancer Risk and Mortality in adults with obesity. JAMA. 2022;327(24):2423–33. 35657620 10.1001/jama.2022.9009 PMC9166218

[6] De Luca M, Shikora S, Eisenberg D, Angrisani L, Parmar C, Alqahtani A, et al. Scientific evidence for the updated guidelines on indications for metabolic and bariatric surgery (IFSO/ASMBS). Surgery for Obesity and Related Diseases. 2024. 10.1007/s11695-024-07370-7 PMC11541402 39320627

[7] Zarshenas N, Tapsell LC, Neale EP, Batterham M, Talbot ML. The relationship between bariatric surgery and Diet Quality: a systematic review. Obes Surg. 2020;30(5):1768–92. 31940138 10.1007/s11695-020-04392-9

[8] Evenepoel C, Vandermeulen G, Luypaerts A, Vermeulen D, Lannoo M, Van der Schueren B, et al. The impact of bariatric surgery on macronutrient malabsorption depends on the type of procedure. Front Nutr. 2023;9:1028881. 36712518 10.3389/fnut.2022.1028881 PMC9877414

[9] Heusschen L. Winning by losing? Nutritional consequences of bariatric surgery. Wageningen University; 2023.

[10] Berrington de Gonzalez A, Hartge P, Cerhan JR, Flint AJ, Hannan L, MacInnis RJ, et al. Body-mass index and mortality among 1.46 million white adults. N Engl J Med. 2010;363(23):2211–9. 21121834 10.1056/NEJMoa1000367 PMC3066051

[11] Alyahya RA, Alnujaidi MA. Prevalence and outcomes of Depression after bariatric surgery: a systematic review and Meta-analysis. Cureus. 2022;14(6):e25651. 35784972 10.7759/cureus.25651 PMC9249077

[12] Franco JVA, Ruiz PA, Palermo M, Gagner M. A review of studies comparing three laparoscopic procedures in bariatric surgery: Sleeve Gastrectomy, Roux-en-Y gastric bypass and adjustable gastric banding. Obes Surg. 2011;21(9):1458–68. 21455833 10.1007/s11695-011-0390-5

[13] Plamper A, Lingohr P, Nadal J, Rheinwalt KP. Comparison of mini-gastric bypass with sleeve gastrectomy in a mainly super-obese patient group: first results. Surg Endosc. 2017;31(3):1156–62. 27444823 10.1007/s00464-016-5085-5

[14] Kabir A, Izadi S, Mashayekhi F, Shokraee K, Rimaz S, Ansar H, et al. Effect of different bariatric surgery methods on metabolic syndrome in patients with severe obesity. Updates Surg. 2024;76(2):547–54. 38051454 10.1007/s13304-023-01699-x

[15] Olbers T, Björkman S, Lindroos A, Maleckas A, Lönn L, Sjöström L, Lönroth H. Body composition, dietary intake, and energy expenditure after laparoscopic Roux-en-Y gastric bypass and laparoscopic vertical banded gastroplasty: a randomized clinical trial. Ann Surg. 2006;244(5):715–22. 17060764 10.1097/01.sla.0000218085.25902.f8 PMC1856598

[16] Mirmiran P, Esfahani FH, Mehrabi Y, Hedayati M, Azizi FJP. Reliability and relative validity of an FFQ for nutrients in the Tehran lipid and glucose study. 2010;13(5):654–62. 10.1017/S1368980009991698 19807937

[17] Ejtahed HS, Sarsharzadeh MM, Mirmiran P, Asghari G, Yuzbashian E, Azizi F. Leemoo, a dietary assessment and nutritional planning software, using fuzzy logic. Int J Endocrinol Metabolism. 2013;11(4):e10169. 10.5812/ijem.10169 PMC3968974 24719623

[18] Ransom D, Ashton K, Windover A, Heinberg LJSO, Diseases R. Internal consistency and validity assessment of SCL-90-R for bariatric surgery candidates. 2010;6(6):622–7. 10.1016/j.soard.2010.02.039 20627709

[19] Ainsworth BE, Haskell WL, Whitt MC, Irwin ML, Swartz AM, Strath SJ et al. Compendium of physical activities: an update of activity codes and MET intensities. 2000;32(9; SUPP/1):S498–504. 10.1097/00005768-200009001-00009 10993420

[20] Kular K, Manchanda N, Rutledge R. Analysis of the five-year outcomes of sleeve gastrectomy and mini gastric bypass: a report from the Indian sub-continent. Obes Surg. 2014;24:1724–8. 24805912 10.1007/s11695-014-1264-4

[21] Wang F-G, Yu Z-P, Yan W-M, Yan M, Song M-M. Comparison of safety and effectiveness between laparoscopic mini-gastric bypass and laparoscopic sleeve gastrectomy: a meta-analysis and systematic review. Medicine. 2017;96(50):e8924. 29390281 10.1097/MD.0000000000008924 PMC5815693

[22] Das K, Nadeem F, Kabir SA. Comparison of one-year outcomes in Sleeve Gastrectomy vs. one anastomosis gastric bypass in a single bariatric unit. Cureus. 2024;16(11):e74838. 39618731 10.7759/cureus.74838 PMC11608598

[23] Calbet JA, Ponce-González JG, Calle-Herrero JdL, Perez-Suarez I, Martin-Rincon M, Santana A et al. Exercise preserves lean mass and performance during severe energy deficit: the role of exercise volume and dietary protein content. 2017;8:483. 10.3389/fphys.2017.00483 PMC5522839 28790922

[24] Celis-Morales CA, Petermann F, Steell L, Anderson J, Welsh P, Mackay DF et al. Associations of dietary protein intake with fat-free mass and grip strength: a cross-sectional study in 146,816 UK Biobank participants. 2018;187(11):2405–14. 10.1093/aje/kwy134 29961893

[25] Baad V, Bezerra LR, de Holanda NC, Dos Santos AC, da Silva AA, Bandeira F, et al. Body composition, sarcopenia and physical performance after bariatric surgery: differences between sleeve gastrectomy and Roux-En-Y gastric bypass. Obesity Surgery. 2022;32(12):3830–8. 10.1007/s11695-022-06335-y 36303085

[26] Kansou G, Lechaux D, Delarue J, Badic B, Le Gall M, Guillerm S, et al. Laparoscopic sleeve gastrectomy versus laparoscopic mini gastric bypass: one year outcomes. Int J Surg. 2016;33:18–22. 10.1016/j.ijsu.2016.07.051 27452299

[27] Thereaux J, Corigliano N, Poitou C, Oppert JM, Czernichow S, Bouillot JL. Comparison of results after one year between sleeve gastrectomy and gastric bypass in patients with BMI ≥ 50 kg/m². Surg Obes Relat Diseases: Official J Am Soc Bariatr Surg. 2015;11(4):785–90. 10.1016/j.soard.2014.11.022 25771441

[28] Faria SL, Faria OP, Lopes TC, Galvão MV, de Oliveira Kelly E, Ito MKJOs. Relation between carbohydrate intake and weight loss after bariatric surgery. 2009;19(6):708–16. 10.1007/s11695-008-9583-y 18618210

[29] Janmohammadi P, Sajadi F, Alizadeh S, Daneshzad EJO. Comparison of energy and food intake between gastric bypass and sleeve gastrectomy: a meta-analysis and systematic review. 2019;29(3):1040–8. 10.1007/s11695-018-03663-w 30610675

[30] Ormancı N, Mercanlıgil MS, Esatoglu V. The Effect of Bariatric Surgery on Nutritional Status and Biochemical Parameters: A Longitudinal Study. 2023.

[31] Ruiz-Tovar J, Bozhychko M, Del-Campo JM, Boix E, Zubiaga L, Muñoz JL, Llavero C. Changes in frequency intake of foods in patients undergoing sleeve gastrectomy and following a strict dietary control. Obes Surg. 2018;28:1659–64. 29250751 10.1007/s11695-017-3072-0

[32] Abrahamse WJFip. How to effectively encourage sustainable food choices: a mini-review of available evidence. 2020;11:589674. 10.3389/fpsyg.2020.589674 PMC7701282 33304299

[33] Stefater MA, Wilson-Pérez HE, Chambers AP, Sandoval DA, Seeley RJ. All bariatric surgeries are not created equal: insights from mechanistic comparisons. Endocr Rev. 2012;33(4):595–622. 22550271 10.1210/er.2011-1044 PMC3410227

[34] Andreu A, Moizé V, Rodríguez L, Flores L, Vidal JJO. Protein intake, body composition, and protein status following bariatric surgery. 2010;20(11):1509–15. 10.1007/s11695-010-0268-y 20820937

[35] Aguas-Ayesa M, Yárnoz-Esquíroz P, Olazarán L, Gómez-Ambrosi J, Frühbeck G. Precision nutrition in the context of bariatric surgery. Reviews Endocr Metabolic Disorders. 2023;24(5):979–91. 10.1007/s11154-023-09794-5 PMC10020075 36928810

[36] Herpertz S, Kielmann R, Wolf A, Langkafel M, Senf W, Hebebrand J. Does obesity surgery improve psychosocial functioning? A systematic review. Int J Obes. 2003;27(11):1300–14. 10.1038/sj.ijo.0802410 14574339

[37] Griauzde DH, Ibrahim AM, Fisher N, Stricklen A, Ross R, Ghaferi AA. Understanding the psychosocial impact of weight loss following bariatric surgery: a qualitative study. BMC Obes. 2018;5:1–9. 30524743 10.1186/s40608-018-0215-3 PMC6276134

[38] deMeireles AJ, Carlin AM, Bonham AJ, Cassidy R, Ross R, Stricklen A, et al. A longitudinal analysis of variation in psychological well-being and body image in patients before and after bariatric surgery. Ann Surg. 2020;271(5):885–90. 30688686 10.1097/SLA.0000000000003146

[39] Behrens SC, Lenhard K, Junne F, Ziser K, Lange J, Zipfel S, et al. Effects of bariatric surgery on depression: role of body image. Obes Surg. 2021;31:1864–8. 33089383 10.1007/s11695-020-05057-3 PMC8012322

[40] Pyykkö JE, Zwartjes M, Nieuwdorp M, van Olst N, Bruin SC, van de Laar AW, et al. Differences in Psychological Health and Weight Loss after bariatric metabolic surgery between patients with and without Pain syndromes. Obes Surg. 2024;34(5):1693–703. 38499942 10.1007/s11695-024-07171-y PMC11031447

[41] Hamamah S, Hajnal A, Covasa M. Influence of bariatric surgery on gut microbiota composition and its implication on brain and peripheral targets. Nutrients. 2024;16(7):1071. 38613104 10.3390/nu16071071 PMC11013759

[42] Drucker DJ. Mechanisms of action and therapeutic application of glucagon-like peptide-1. Cell Metabol. 2018;27(4):740–56. 10.1016/j.cmet.2018.03.001 29617641

[43] Müller T, Finan B, Clemmensen C, DiMarchi R, Tschöp M. The new biology and pharmacology of glucagon. Physiol Rev. 2017;97(2):721–66. 28275047 10.1152/physrev.00025.2016

[44] Labarthe A, Fiquet O, Hassouna R, Zizzari P, Lanfumey L, Ramoz N, et al. Ghrelin-derived peptides: a link between appetite/reward, GH axis, and psychiatric disorders? Front Endocrinol. 2014;5:163. 10.3389/fendo.2014.00163 PMC4209873 25386163

[45] Al-Kadi A, Al-Sulaim L. Psychological and social outcomes of patients following bariatric surgery: a systematic review. Pol J Surg. 2022;96(SUPLEMENT 1):53–9. 10.5604/01.3001.0016.1104 36808065

[46] Auer CJ, Glombiewski JA, Doering BK, Winkler A, Laferton JA, Broadbent E, Rief W. Patients’ expectations predict surgery outcomes: a meta-analysis. Int J Behav Med. 2016;23:49–62. 26223485 10.1007/s12529-015-9500-4

[47] Martens K, Hamann A, Miller-Matero LR, Miller C, Bonham AJ, Ghaferi AA, Carlin AM. Relationship between depression, weight, and patient satisfaction 2 years after bariatric surgery. Surg Obes Relat Dis. 2021;17(2):366–71. 33127323 10.1016/j.soard.2020.09.024

